# Performance evaluation of Espline HTLV-I/II, a newly developed rapid immunochromatographic antibody test for different diagnostic situations

**DOI:** 10.1128/spectrum.02078-23

**Published:** 2023-11-15

**Authors:** Madoka Kuramitsu, Haruka Momose, Yuichiro Uchida, Kenji Ishitsuka, Ryuji Kubota, Masahito Tokunaga, Atae Utsunomiya, Kunihiko Umekita, Yuuki Hashikura, Kisato Nosaka, Ki-Ryang Koh, Hitomi Nakamura, Yasuko Sagara, Rieko Sobata, Masahiro Satake, Koh Nagata, Yuri Hasegawa, Daisuke Sasaki, Hiroo Hasegawa, Tomoo Sato, Yoshihisa Yamano, Kou Hiraga, Kenta Tezuka, Emi Ikebe, Sahoko Matsuoka, Kazu Okuma, Toshiki Watanabe, Kiyonori Miura, Isao Hamaguchi

**Affiliations:** 1 Research Center for Biological Products in the Next Generation, National Institute of Infectious Diseases, Tokyo, Japan; 2 Department of Hematology and Rheumatology, Graduate School of Medical and Dental Sciences, Kagoshima University, Kagoshima, Japan; 3 Division of Neuroimmunology, Joint Research Center for Human Retrovirus Infection, Kagoshima University, Kagoshima, Japan; 4 Department of Hematology, Imamura General Hospital, Kagoshima, Japan; 5 Division of Respirology, Rheumatology, Infectious Diseases and Neurology, Department of Internal Medicine, University of Miyazaki, Miyazaki, Japan; 6 Department of Clinical Laboratory, University of Miyazaki Hospital, Miyazaki, Japan; 7 Cancer Center, Kumamoto University Hospital, Kumamoto, Japan; 8 Department of Hematology, Osaka General Hospital of West Japan Railway Company, Osaka, Japan; 9 Japanese Red Cross Kyushu Block Blood Center, Fukuoka, Japan; 10 Central Blood Institute, Blood Service Headquarters, Japanese Red Cross Society, Tokyo, Japan; 11 Department of Obstetrics and Gynecology, Nagasaki University Graduate School of Biomedical Sciences, Nagasaki, Japan; 12 Department of Laboratory Medicine, Nagasaki University Hospital, Nagasaki, Japan; 13 Department of Neurology, St. Marianna University School of Medicine, Kanagawa, Japan; 14 Department of Rare Diseases Research, Institute of Medical Science, St. Marianna University School of Medicine, Kanagawa, Japan; 15 Department of Microbiology, Faculty of Medicine, Kansai Medical University, Osaka, Japan; 16 Laboratory of Practical Management of Medical Information, Graduate School of Medicine, St. Marianna University, Kanagawa, Japan; 17 Department of Clinical Laboratory, Subaru Health Insurance Society Ota Memorial Hospital, Gunma, Japan; Kumamoto Daigaku, Kumamoto, Japan

**Keywords:** immunochromatographic antibody test, point-of-care test, HTLV-1, sensitivity, specificity

## Abstract

Antibody screening tests for human T-cell leukemia virus type 1 (HTLV-1) are performed based on methods such as chemiluminescent enzyme immunoassay (CLEIA), chemiluminescence immunoassay (CLIA), electrochemiluminescence immunoassay, and particle agglutination (PA). Espline HTLV-I/II, a commercially available, easy-to-use, and rapid immunochromatographic antibody test (IC), was developed for situations where expensive instruments and laboratory equipment are not available. In this report, we compared the performance of IC with the above existing tests using diverse samples derived from asymptomatic HTLV-1 carriers and patients with HTLV-1-associated diseases in collaboration with 11 Japanese institutes. We found that IC detected HTLV-1 infection in all samples from HTLV-1-associated diseases, including adult T-cell leukemia, HTLV-1-associated myelopathy, and HTLV-1 uveitis (200/200). The sensitivity of IC compared with CLIA, CLEIA, and PA was 99.2% (363/366), 100% (241/241), and 100% (47/47), respectively, and the specificity was 99.4% (994/1000), 100% (60/60), and 100% (40/40), respectively. The positive and negative predictive values of IC were 99.7% [95% confidence interval (CI): 99.12–99.92] and 99.5% (95% CI: 98.82–99.75), respectively. However, IC had difficulty in correctly judging samples that were diagnosed as seroreactive in other first screening tests but negative by a confirmatory test; for example, of 612 confirmed negative samples that were CLIA seroreactive, 332 samples were IC positive. These results confirmed that IC has sufficient sensitivity and specificity as a screening test for HTLV-1, although, like the other screening tests, it also requires a confirmatory test to determine HTLV-1 infection correctly.

**IMPORTANCE:**

The World Health Organization estimated that 5–10 million people are infected with human T-cell leukemia virus type 1 (HTLV-1). This number is likely to be underestimated because reliable endemic data are available for only approximately 1.5 billion people worldwide. The point-of-care test is a powerful tool for the easy and quick detection of infections without the requirement for expensive instruments and laboratory equipment. Espline HTLV-I/II, a newly developed rapid immunochromatographic antibody test that was evaluated in this study, might significantly advance our understanding of the global epidemiology of HTLV-1 infection.

## INTRODUCTION

Human T-cell leukemia virus type 1 (HTLV-1) is a retrovirus that causes a life-long infection by integrating its proviral genome into the host genome (1). HTLV-1 is endemic in southwestern Japan, sub-Saharan Africa, the Caribbean, South America, the Middle East, and Australo-Melanesia (2). The World Health Organization estimated that 5–10 million people are infected with HTLV-1 (3). This number is likely to be an underestimate because reliable endemic data are available for only approximately 1.5 billion people worldwide (2). The main routes of infection are mother-to-child transmission, sexual intercourse, and transfusion of unscreened blood. Among infected people, small numbers develop adult T-cell leukemia/lymphoma (ATL), HTLV-1-associated myelopathy (HAM), or HTLV-1 uveitis (HU) (4 – 6). In addition, kidney transplantation from an HTLV-1-positive donor to a negative recipient develops HAM at a high rate (40%) with a shorter incubation period (7).

HTLV-2 is another retrovirus that is closely related to HTLV-1 (8). HTLV-1 and HTLV-2 are the most prevalent types in this virus group. HTLV-2 is endemic in North and South America, Europe, and Central Africa (9 – 11). Although previous reports suggested a correlation between HTLV infection and hairy cell leukemia or neurological disorders (8, 12), HTLV-2 pathogenicity is not fully understood.

Diagnostic methods for HTLV-1 or HTLV-1/2 antibody screening such as chemiluminescent enzyme immunoassay (CLEIA), chemiluminescence immunoassay (CLIA), electrochemiluminescence immunoassay (ECLIA), and particle agglutination (PA) are available (13 – 15). CLEIA, CLIA, and ECLIA are based on third-generation double antigen sandwich methods that achieve high specificity by the elimination of nonspecific reactions caused by secondary antibodies. Because false-positive results occur when using these methods, seroreactive results should be confirmed by a line immunoassay (LIA) or western blotting (WB) (16, 17). In addition, PCR with HTLV-1/2-specific primer-probes is used to confirm indeterminate results obtained by LIA or WB (16, 18).

A point-of-care test is a powerful tool for the easy and rapid detection of infections without the requirement for expensive instruments and laboratory equipment; however, no serological point-of-care test kits for HTLV-1 and HTLV-2 are commercially available worldwide.

Espline HTLV-I/II, an immunochromatographic antibody test (IC), was developed as a novel point-of-care test kit for the diagnosis of HTLV-1 infection and has been authorized as an *in vitro* diagnostic kit in Japan since 2023. This kit is based on the detection of a serological reaction between specific human IgG and recombinant HTLV-1 gp21 antigen, and the synthesized peptides HTLV-1 gp46 and HTLV-2 gp46, in a lateral flow assay.

In this study, we collaborated with 11 Japanese institutes, and collected sera or plasma samples from patients with HTLV-1-associated diseases, outpatients, pregnant women, and voluntary blood donors, and the sensitivity and specificity of Espline HTLV-I/II were evaluated. Moreover, of the collected samples, cases with a discrepancy between screening and confirmatory tests were examined for precise evaluation. Throughout the evaluation, the performance of Espline HTLV-I/II in diverse HTLV-1 diagnostic situations was confirmed.

## RESULTS

### Analysis of the sensitivity of IC

Overall, 2,855 samples (1,511 men, 1,164 women, median age 47, sex and age not available for 160) were enrolled. Out of these samples, 1,115 were confirmed positive for HTLV-1 and 1,740 were confirmed negative for HTLV-1. The antibody test methods used in this study are listed in Table 1. As shown in Table 2, IC indicated seroreactivity for all HTLV-1-associated diseases including ATL (100%, 73/73), HAM (100%, 126/126), and HU (1/1), regardless of the type of first screening test. Additionally, IC correctly detected 100% of asymptomatic carriers who were seropositive, which were screened by PA and CLEIA. Among 296 CLIA+/LIA+ samples, 1.0% (3/296) were IC negative, which is inconsistent with the final judgment as HTLV-1 seropositive. The specificity of IC to PA, CLEIA, and CLIA was statistically calculated as 100% [95% confidence interval (CI): 92.44–100], 100% (95% CI: 96.24–100), and 99.2% (95% CI: 97.64–99.78), respectively (Table 3).

**TABLE 1:** Diagnostic kits used in this study

Name of kit	Method	Company	Kit name in Table 2
Lumipulse Presto HTLV-I/II	CLEIA	Fujirebio Inc.	CLEIA A
HISCL HTLV-I Ab	CLEIA	Sysmex Corporation	CLEIA B
Lumipulse Presto HTLV-I	CLEIA	Fujirebio Inc.	CLEIA C
Lumipulse HTLV-I/II	CLEIA	Fujirebio Inc.	CLEIA D
Architect HTLV-I/II Score	CLIA	Abbott Japan LLC	CLIA
Serodia HTLV-I	PA	Fujirebio Inc.	PA
Problot HTLV-I	WB	Fujirebio Inc.	WB
INNO-LIA HTLV-I/II Score	LIA	Fujirebio Inc.	LIA

**TABLE 2:** Performance evaluation of Espline HTLV-I/II for different diagnostic situations

HTLV-1 diagnosis	Clinical symptom	First antibody test	Confirmatory test	Additional test	Number of samples	IC positive	IC negative
Positive	ATL	CLEIA A+	LIA+	N/A^*a*^	43	43	0
		CLIA+	LIA+	N/A	30	30	0
	HAM	CLEIA A+	LIA+	N/A	1	1	0
		CLEIA B+	LIA+	N/A	85	85	0
		CLIA+	LIA+	N/A	40	40	0
	HU	CLEIA A+	LIA+	N/A	1	1	0
	Asymptomatic	PA+	LIA+	N/A	47	47	0
		CLEIA A+	WB +	N/A	130	130	0
		CLEIA C+	WB+	N/A	70	70	0
		CLEIA A+	LIA+	N/A	66	66	0
		CLEIA B+	LIA+	N/A	68	68	0
		CLEIA C+	LIA+	N/A	23	23	0
		CLEIA D+	LIA+	N/A	99	99	0
		CLIA+	LIA+	N/A	296	293	3
		CLEIA A+	WB indeterminate	PCR+	47	47	0
		CLEIA C+	WB indeterminate	PCR+	53	53	0
		CLEIA C+	LIA indeterminate	PCR+	2	1	1
		CLEIA D+	LIA indeterminate	PCR+	1	1	0
		CLIA+	LIA indeterminate	PCR+	13	11	2
Negative	Healthy	PA−	N/A	N/A	40	0	40
		CLEIA D−	N/A	N/A	60	0	60
		CLIA−	N/A	N/A	1,000	6	994
		CLEIA B+	LIA−	N/A	19	0	19
		CLEIA C+	LIA−	N/A	4	1	3
		CLIA+	LIA−	N/A	451	205	246
		CLEIA A+	LIA indeterminate	PCR−	1	1	0
		CLEIA C+	LIA indeterminate	PCR−	4	0	4
		CLIA+	LIA indeterminate	PCR−	161	127	34

^*a*^ N/A, not applicable.

**TABLE 3:** Statistical analysis of the sensitivity and specificity of Espline HTLV-I/II with a 95% confidence interval

Assay name	Method	Sensitivity (95% CI)	Specificity (95% CI)
Serodia HTLV-I	PA	100% (92.44–100)	100% (91.24–100)
Lumipulse HTLV-I/II	CLEIA	100% (96.26–100)	100% (93.98–100)
Architect HTLV-I/II Score	CLIA	99.19% (97.64–99.78)	99.40% (98.70–99.72)

Furthermore, IC correctly detected 100% of WB indeterminate/PCR-positive samples, indicating that IC did not miss these provirus-positive samples as a first screening test. In addition, as shown in Table 2, IC detected infections in 81.3% (13/16) of samples that were LIA indeterminate and PCR positive. Among these 16 samples, 13 had IC and PA results available. As shown in Table 4, of the 13 cases, 8 were seroreactive by IC and PA. Cases 4, 6, 7, and 12 showed inconsistent results between the tests. In particular, cases 4 and 6 were only seroreactive by IC and showed gp21 single bands by LIA, suggesting differences in the sensitivity to gp21 protein. By contrast, case 12 was only seroreactive by PA, showed only Gag bands by LIA, and was seronegative by CLIA, suggesting the low titer of anti-gp21 antibodies. These results indicate that IC had a similar sensitivity to PA when using samples that were difficult to confirm as seropositive.

**TABLE 4:** Performance of Espline HTLV-I/II with LIA indeterminate provirus-positive samples^*e*^

No.	CLIA, S/CO^*a*^	ECLIA, S/CO	CLEIA^*b*^, COI^*c*^	PA	IC	LIA	LIA band intensity							PCR
							p19	p24	gp46	gp21	p19-I	gp46-I	gp46-II	
1	34.6	16.6	NT^*d*^	+	+	Indeterminate	−	−	−	2+	−	−	−	+
2	36.6	12.3	NT	+	+	Indeterminate	−	−	−	2+	−	−	−	+
3	11.0	4.4	NT	+	+	Indeterminate	−	−	−	2+	−	−	−	+
4	2.1	1.3	NT	−	+	Indeterminate	−	−	−	2+	−	−	−	+
5	3.5	11.6	NT	+	+	Indeterminate	−	−	−	±	−	−	−	+
6	3.7	4.4	NT	−	+	Indeterminate	−	−	−	±	−	−	−	+
7	2.2	4.2	NT	+	−	Indeterminate	−	−	−	±	−	−	−	+
8	1.9	99.6	NT	−	−	Indeterminate	−	−	−	1+	−	−	−	+
9	4.2	44.5	NT	+	+	Indeterminate	−	−	−	2+	−	−	−	+
10	21.1	38.8	NT	+	+	Indeterminate	−	−	−	2+	−	−	−	+
11	7.61	NT	NT	+	+	Indeterminate	2+	2+	−	−	−	−	−	+
12	0.16	NT	2.7	+	−	Indeterminate	1+	±	−	−	−	−	−	+
13	NT	NT	2.3	+	+	Indeterminate	−	−	−	1+	−	−	−	+

^*a*^ S/CO, signal-to-cutoff ratio.

^*b*^ CLEIA, Lumipulse Presto HTLV-I.

^*c*^ COI, cutoff index.

^*d*^ NT, not tested.

^*e*^ Underlined numbers are scores that were applied at the antibody screening test.

### Analysis of the specificity of IC

Next, the specificity of IC was evaluated using samples that were not reactive at the first screening. Importantly, the results of IC were 100% when using PA and CLEIA, and 99.4% of the results matched those of CLIA (of 1,000 samples, 6 were seroreactive by IC) (Table 2). The sensitivity of IC to PA, CLEIA, and CLIA was statistically calculated as 100% (95% CI: 91.24–100), 100% (95% CI: 93.98–100), and 99.4% (95% CI: 98.70–99.72), respectively (Table 3).

Notably, there was a significant number of inconsistent results for samples that were seroreactive at first screening but negative for LIA (43.5%, 206/474) or were LIA indeterminate and PCR negative (77.1%, 128/166) (Table 2).

### Analysis of inconsistent results between IC and confirmatory tests

To analyze the inconsistency between the results of IC and the existing kits, we focused on three samples that were HTLV-1 positive and IC negative (Table 2). The intensity of these samples in CLIA was extremely low and close to the cutoff values (Table 5). Furthermore, six HTLV-1 samples that were negative by CLIA were seroreactive by IC (Table 2). These samples were tested by LIA and PCR, and five of the six samples were negative and one was indeterminate by LIA (data not shown). PCR was used to test the indeterminate sample, but no proviral DNA was detected (data not shown). Therefore, six of the samples indicated to be seroreactive by IC were confirmed as false positives.

**TABLE 5:** Analysis of antibody titers of IC negative and HTLV-1 confirmed positive samples

No.	IC	CLIA, S/CO	LIA								PCR
			Result	p19	p24	gp46	gp21	p19-I	gp46-I	gp46-II	
1	−	1.2	HTLV	±	−	−	±	−	−	−	UD^*a*^
2	−	1.0	HTLV	±	±	−	+	−	−	−	UD
3	−	1.4	HTLV	1+	±	−	±	−	−	−	UD

^*a*^ UD, undetectable.

## DISCUSSION

An estimated 5–10 million people are infected with HTLV-1, but this number is calculated from limited epidemiological data based on only 1.5 billion people (19). One limitation regarding worldwide epidemiology is the lack of available tests in low-resource countries. For example, sub-Saharan Africa is one of the largest HTLV-1 endemic regions, and among the countries in this region, the central African countries of Gabon and Cameroon have been extensively studied regarding HTLV-1 prevalence. The prevalence of HTLV-1 was estimated to be 5%–15% in Gabon (20, 21) and 1%–2% in Cameroon (22, 23). However, data remain limited for the estimation of overall prevalence of HTLV-1 in African countries (19). A point-of-care test does not require expensive instruments or laboratory equipment and would enhance our understanding of the epidemiology of HTLV-1 infection globally. Thus, reliable point-of-care tests are urgently needed for HTLV-1 diagnosis.

Antigen double sandwich enzyme-linked immunosorbent assay methods for HTLV-1 antibody tests show high performance in terms of HTLV-1 diagnosis. The Architect rHTLV-I/II assay showed 100% sensitivity and 99.98% specificity with previously diagnosed samples, and the results completely matched with the Murex HTLV-I/II test (14). Similarly, high sensitivities (99.4% and 100%) and specificities (100% and 99.98%) for the Architect rHTLV-I/II assay were reported by two other research groups (14, 24). The Elecsys HTLV-I/II assay showed 100% sensitivity and 99.91% specificity to the Architect rHTLV-I/II assay and 98.63% specificity and 99.95% specificity to the Ortho Avioq HTLV-I/II assay using a large-scale collection of samples from Europe and Japan (13). In addition, both the Abbott Alinity i rHTLV-I/II and DiaSorin Liaison XL murex recHTLV-I/II tests showed 100% sensitivity and 100% specificity to the Architect rHTLV-I/II assay (25).

In this study, the performance of a newly developed IC, Espline HTLV-I/II, was evaluated using samples obtained under various conditions during the diagnosis of HTLV-1 infection. In particular, IC had 100% sensitivity for cases with HTLV-1-associated diseases ATL, HAM, and HU. Extremely high sensitivities were also observed for samples from HTLV-1 carriers regardless of the type of screening kit used. Similar high specificity performance was also observed when screening HTLV-1-negative samples. The sensitivity and specificity of IC were thought to be equivalent to the high performance HTLV-1 antibody tests described above. The confidence interval (95%) of IC overlapped with those obtained with the PA, CLEIA, and CLIA methods (Table 3). These results suggested that IC might be useful for the screening of HTLV-1 infection, similar to the currently used sandwich or conventional methods, such as CLEIA, CLIA, and PA.

Our results also suggested the usefulness of IC in clinics or situations where expensive automated instruments for antibody tests are not available. However, IC had a high rate of false positives for samples that were seroreactive at screening but confirmed negative by LIA or PCR. Thus, it is important that diagnoses are not made using IC positive results alone. This concern may be addressed by updating the diagnostic flow of the determination of HTLV-1 infection by incorporating IC as a screening test.

HTLV-1 gp21 is a prominent antigen for the detection of HTLV-1 infection (16, 26). Even though IC and LIA tests commonly use the HTLV-1 gp21 antigen, the specificities of these tests were different in our study. One explanation might be differences in the protocols for the IC and LIA tests, such as incubation time and wash steps. These differences might increase the specificity of LIA by increasing specific binding and decreasing the nonspecific binding of antibodies.

Because the IC detection system is based on an antibody–antigen binding reaction, it might provide a negative result for samples with low antibody titers, such as those obtained at the early stage of infection. As shown in this study, IC did not correctly identify infection in a small number of seropositive samples with antibody titers close to the assay cutoff level. A previous study estimated a window period of at least 2.2 ± 0.6 months for the serological detection of HTLV-1 using HTLV-1 screening tests (27). Although the optimum period for the serological detection of HTLV-1 by IC has not been estimated, a person who might be within this period should be followed and reexamined several months later.

Because of the limitation of available samples, this study was performed on Japanese samples only. The phylogenetic background of the majority of samples was thought to be subtype A and the transcontinental and Japanese subgroups. The performance of IC with samples from other large endemic areas, such as the regions with subtypes B and D in Africa or subtype C in Australia, and against HTLV-2 should be evaluated further. In addition, because we could only use the first screening kits licensed in Japan, we could not evaluate the performance of IC with other previously reported screening kits. Furthermore, the majority of enrolled samples were screened by CLIA, and the characteristics of the enrolled samples were possibly influenced by the performance of the first screening kit.

In conclusion, this study confirmed that Espline HTLV-I/II is a reliable HTLV-1 screening test.

## MATERIALS AND METHODS

### Study design

The performance of IC was evaluated using the serum or plasma samples that were previously diagnosed by HTLV-1 screening tests and confirmatory tests. Samples were obtained collaboratively from 11 Japanese institutes. The storage temperature of samples was below −20°C. The diagnostic criteria for HTLV-1 infection were based on guidelines for HTLV-1 infection in Japan (16). Precise information about sample size and the collection time period, as well as the sex, age, and clinical symptoms of participants, is listed in Tables S1 and S2. For comparison with the IC results, the results of other screening tests and confirmatory tests were used at the point of diagnosis of HTLV-1 infection retrospectively. Generally, IC was performed at each institute, but in some cases where this was not possible, IC was performed at the National Institute of Infectious Diseases. To evaluate the performance of IC with samples that showed a discrepancy between the screening test and confirmatory test, first screening positive but WB indeterminate, LIA indeterminate, and LIA-negative samples were included. In addition, PCR results were used as a confirmatory test for indeterminate samples. WB indeterminate, LIA indeterminate, and LIA-negative samples were excluded from the analyses of sensitivity and specificity.

### Clinical samples

HTLV-1-positive and HTLV-1-negative sera or plasma were obtained with informed consent from patients with HTLV-1-associated diseases including ATL, HAM, and HU, asymptomatic carriers, pregnant women, blood donors, and from residual samples obtained during general clinical practice.

### Antibody test

Espline HTLV-I/II, an IC for HTLV-I/II, was kindly provided by Fujirebio Inc. (Tokyo, Japan). To confirm HTLV-1 infection, initial screening tests were performed by PA, CLIA, or CLEIA (Table 1). Secondary confirmatory tests were performed by LIA or WB (Table 1). The antibody test kits used in this study are described in Table 1. Indeterminate results from a secondary test were tested by HTLV-1 PCR as reported previously (28 – 32). ECLIA (Elecsys HTLV-I/II assay, Roche Diagnostics K.K., Tokyo, Japan) was additionally performed for samples that showed indeterminate patterns by LIA with a detectable level of HTLV-1 provirus by PCR. Results were double checked by another researcher at each institute. For the confirmation of results with discrepancy between IC and other tests, IC was retested on a different day and the reproducibility of IC was confirmed.

### Analysis of the sensitivity and specificity of IC

The sensitivities and specificities of IC to other screening tests with a 95% confidence interval were statistically calculated by bivariate analysis with the *χ* ^2^ test using the GraphPad Prism 7 software (GraphPad, San Diego, CA, USA). Overall positive and negative productive values of IC were also statistically calculated by bivariate analysis with *χ* ^2^ test using GraphPad Prism 7.

## Data Availability

The findings reported here are available from the corresponding author upon request.

## SUPPLEMENTAL MATERIAL

Tables S1 and S2 spectrum.02078-23-s0001.docx Clinical symptoms, sample size, and the sex and age of samples included in this study.

ASM does not own the copyrights to Supplemental Material that may be linked to, or accessed through, an article. The authors have granted ASM a non-exclusive, world-wide license to publish the Supplemental Material files. Please contact the corresponding author directly for reuse.
