# Supplementary material for: Performance evaluation of Espline HTLV-I/II, a newly developed rapid immunochromatographic antibody test for different diagnostic situations
Source: Microbiol Spectr. 2023 Nov 15;11(6):e02078-23. doi: 10.1128/spectrum.02078-23 (PMC10715220; doi:10.1128/spectrum.02078-23)
Supplement: Tables S1 and S2 — Clinical symptoms, sample size, and the sex and age of samples included in this study. [file spectrum.02078-23-s0001.docx]

Supplemental Data

Supplemental Table S1. Preparation of samples from Japanese institutes for the evaluation of IC

| Institute | Number of samples | Sex | | Age - median | Age - range | Place of study | Clinical symptoms | Date of collection |
| --- | --- | --- | --- | --- | --- | --- | --- | --- |
|  |  | Men | Women |  |  |  |  |  |
| A | 40 | 10 | 30 | 61 | 29 – 84 | A | ATL, AC | 2012.7 – 2020.8 |
| B | 50 | 23 | 27 | 65 | 31 – 79 | B | ATL, HAM, AC | N.A. |
| C | 20 | 6 | 14 | 65 | 38 – 87 | C | ATL, HU, AC | 2021.6 – 2021.12 |
| D | 50 | 7 | 43 | 66 | 32 – 87 | D | HAM, AC | N.A. |
| E | 172 | 44 | 128 | 58 | 25 – 83 | NIID | HAM, AC, Negative | 2011.11 – 2020.10 |
| F | 87 | 0 | 87 | 31 | 22 – 44 | NIID | AC, Negative | 2012 (AC), 2021 (Negative) |
| G | 50 | 18 | 32 | 66 | 35 – 84 | NIID | AC, Negative | 2013.3 – 2020.3 |
| H | 1830 | 1181 | 649 | 47 | 16 – 69 | NIID | AC, Negative | 2021.6 – 2021.12 |
| I | 396 | 228 | 168 | 48 | 17 – 69 | NIID | AC, Negative | 2008.8 – 2022.8 |
| J | 160 | N.A. | | N.A. | | J | AC, Negative | 2016.4 – 2021.1 |
| NIID | - | - | - | - | - | - | - | - |

ATL: adult T-cell leukemia, HAM: HTLV-1-associated myelopathy, HU: HTLV-1 uveitis, AC: asymptomatic carrier, N.A.: not applicable, NIID: National Institute of Infectious Diseases.

Supplemental Table S2. Clinical symptoms, sample size, and the sex and age of patients included in this study

| Clinical symptoms | Number of samples | Sex | | Age - median | Age - range | N.A. |
| --- | --- | --- | --- | --- | --- | --- |
|  |  | Men | Women |  |  |  |
| ATL | 73 | 27 | 46 | 66 | 44 – 84 | - |
| HAM | 126 | 29 | 97 | 65 | 34 – 87 | - |
| HU | 1 | 0 | 1 | 71 | - | - |
| AC | 915 | 382 | 423 | 49 | 17 – 84 | 100 |
| Negative | 1740 | 1076 | 604 | 45 | 16 – 72 | 60 |

N.A.: sex and age not applicable.
